# Culture Change and Affectionate Communication in China and the United States: Evidence From Google Digitized Books 1960–2008

**DOI:** 10.3389/fpsyg.2019.01110

**Published:** 2019-05-22

**Authors:** Michael Shengtao Wu, Boyuan Li, Liangliang Zhu, Chan Zhou

**Affiliations:** ^1^School of Journalism and Communication, Xiamen University, Xiamen, China; ^2^School of Information Science and Engineering, Xiamen University, Xiamen, China; ^3^Institute of Developmental Psychology, Beijing Normal University, Beijing, China

**Keywords:** affectionate communication, social change, cultural change, urbanization, Google Ngram Viewer

## Abstract

Humans are born with the ability and the need for affection, but communicating affection as a social behavior is historically bound. Based on the digitized books of Google Ngram Viewer from 1960 through 2008, the present research investigated affectionate communication (AC) in China and the United States, and its changing landscape along with social changes from collectivist to individualistic environments. In particular, we analyzed the frequency in terms of verbal affection (e.g., love you, like you), non-verbal affection (e.g., hug, kiss), and individualism (indicated by the use of first-person singular pronouns such as I, me, and myself) in Chinese and American books. The results revealed an increasing trend for AC in recent decades, although the frequency of affection words was lower in Chinese than in American books. Further, individualism was positively related to the frequency of affection words in both Chinese and American books. These results demonstrate the effect of cultural changes on AC, in that affection exchange becomes popular in adaptation to individualistic urban environments. These findings exemplify a cross-cultural difference in the expression of love and the cultural universality of social change in Eastern and Western societies.

## Introduction

The sight of dozens of shoppers passionately kissing each other in a mall would be surprising in any country, but especially so in China where public affection is frowned upon…The competition is to let people in love express themselves and enjoy the moment.

– *Daily Mail, June 8 2010*

The public expression of love toward a spouse or romantic partner has long been discouraged in Asian cultures such as China (Lee, 2007; Kline et al., 2008). This proscription appears to be relaxing, however, with Chinese adults observed kissing and hugging, even in public. Some scholars have argued that this change is due to the exposure to the West, especially expressive North American cultures (Dion and Dion, 1993; Hatfield and Forbes, 2013). In the present research, we propose that shifts from rural community (e.g., subsistence, collectivistic) to urban society (e.g., commercial, individualistic) in China and the United States have further contributed to increases in the acceptance of affectionate behavior.

To investigate changes in affectionate expression from the perspective of social change, we first employed affection exchange theory (AET) and then took into consideration the effects of culture. We propose that both China and the United States have experienced increases in individualistic values consistent with a world-wide shift from agricultural communities to industrial societies.

### Affectionate Communication

Love and affection are fundamental needs of social species such as humans (Harlow and Zimmermann, 1959; Rotter et al., 1972), and they play critical roles both in human wellness (Floyd, 2006) and in developmental psychological processes (e.g., Bowlby, 1953). There are many different ways of expressing love, including kissing and hugging (Acker et al., 1973; Acker and Marlon, 1984). As Floyd and Morman (1998) indicated, affectionate communication (AC), which comprises both non-verbal and verbal expressions, is one of the primary means of conveying love and creating intimacy.

According to AET, a propensity for AC has evolved in humans because of its contributions to survival and reproductive success (Floyd, 2006; Floyd et al., 2015). AC covaries with a variety of individual benefits, including happiness, self-esteem, and both mental and physical health, and it promotes the development and maintenance of pair bonds (Floyd et al., 2005). AET therefore proposes that, compared to less-affectionate individuals, highly affectionate individuals are more likely to have successful relationships, be more socially active, be more intimate, and be more satisfied with their relationships (Floyd, 2002; Floyd and Mikkelson, 2002).

Humans vary, however, in both their propensities for affection and in the behaviors through which they express it (Floyd et al., 2015). One factor that accounts for variation in these outcomes is culture (Morman and Floyd, 2002). For example, North Americans may endorse passionate love beliefs and styles more than Asians, who may endorse more companionate and pragmatic beliefs about love (Kline et al., 2008). Overt demonstrations of affection are often encouraged in expressive, and high-contact Western cultures yet discouraged or even proscribed in less-expressive, low-contact cultures (McDaniel and Andersen, 1998; Inglehart and Kingemann, 2000). In China, for example, Confucianism has exerted a concentrated and continuous influence on Chinese society and lays great emphasis on the regulation of social behavior by the patriarchy, leading to a social norm that discourages the overt display of affection.

### Social Change and Cultural Change

Human cultures, which are often differentiated as individualistic or collectivistic, are adapted to ecological conditions and therefore influenced by these conditions (Greenfield, 2013). Individualism is characterized by valuing one’s independence and prioritizing concerns for personal needs and interests, whereas collectivism is reflected in interdependence and concerns about interpersonal bonds, as well as greater awareness of and responsiveness to the needs of others (Triandis, 1989; Markus and Kitayama, 1991).

To date, one of the most significant ecological trends worldwide is a shift to the urban/gesellschaft environment, which has brought about cultural value change that increases individualism and decreases collectivism (Greenfield, 2009, 2013; Yu et al., 2015). For example, the more frequent use of singular pronouns and less frequent use of plural pronouns in American books has suggested a cultural trend toward greater individualism and a parallel trend toward less collectivism in American culture from 1960 to 2008. Likewise, China has experienced rapid economic development and urbanization in recent decades, during which time the frequency of words reflecting individualism increased and that of words reflecting collectivism either declined or else rose more slowly (Zeng and Greenfield, 2015; Zhou et al., 2018).

In gesellschaft environments, the emphasis is on the experience of the individual. Thus, intra-individual phenomena such as personal perspectives, desires, and feelings – all characteristics of the self – are important (Inglehart and Baker, 2000; Kraus et al., 2012; Manago, 2012). In gemeinschaft environments, in contrast, the emphasis is on the group; thus, what is significant are outward behaviors that can be reacted to by other people. In the psychological-behavioral domain, people are focusing on overt action in gemeinschaft environments (e.g., Childs and Greenfield, 1980), whereas they are more attuned to inner psychological processes in gesellschaft environments (Greenfield and Bruner, 1966; Demuth et al., 2012).

### Changing Landscapes of Affection and Love

From the late 1970s to the present, China and Western Europe were both forced into the current round of individualization through the impact of urbanization and globalization. As one of the most important changes that has occurred in the individualization process, the significance of desire and affection in personal life has grown (Yan, 2003). According to Yan (2011), the rise of individualism is best reflected in the legitimization of desires for intimacy, privacy, freedom, and material comforts as well as in the actual pursuit of these desires. In comparison to the traditional corporate family in which discipline was emphasized, choices were controlled, and emotions were avoided for the sake of efficiency and order, individuals in contemporary families express greater investment in personal happiness. Consequently, the latest generation of village youth has begun to regard individual happiness as just as important as that of the conjugal family (Yan, 2011).

Along with this widespread social change, including reform in China, the intimate relationship between lovers and the expression of love are also evolving from role-focused to individual-focused (Zhu, 2004; Zhong and Cheng, 2014). In China, for instance, traditional marriage is typically arranged by parents and elders and is intended for the two families to unite and to have a son carrying on the family name, rather than to promote personal love between spouses. Youngsters who fall in love freely will be subjected to great resistance (Chang, 2006). However, cultural evolution has begun to promote the idea, particularly among educated youth, that marriage should be based on love and choice rather than on the dictates of parents (Yang, 2014; Zhong and Cheng, 2014). Young people are more open and direct when expressing their feelings to lovers or spouses and pay more attention to their partners’ personal behaviors (Yan, 2012), reflecting a more individualistic than collectivistic orientation (Zhong and Cheng, 2014).

In a word, China has changed from a traditional agricultural to a modern industrial society, and its culture has become increasingly individualized. The individualistic values brought about by urbanization and globalization have indeed influenced people’s views on intimate relationships and the expressions of love. Few studies have investigated the effect of social change on AC thus far, but recent findings have shown that AC benefits Chinese young adults (in the form of higher subjective well-being and more romantic partners; Wu et al., 2014), who traditionally belong to a low-contact culture where expression of love is not encouraged (McDaniel and Andersen, 1998), at levels equivalent to the benefits shared by American young adults.

### The Present Research

In the present study, an online corpus of American and Chinese cultural products (identified by Google Book Ngram Viewer) was used to examine changes in references to AC behaviors – both verbal (e.g., *love you*, *like you*) and non-verbal (e.g., hug, kiss) – during the period from 1960 to 2008 and their relationship to increases in individualism (indicated by the use of first-person singular pronouns such as *I*, *me*, *my*, *mine*, and *myself*).

Due to widespread shifts from rural community/gemeinschaft to urban society/gesellschaft, we expect that the frequency of affection words and expressions will increase from 1960 to 2008 in both American and Chinese books, and that this trend can be accounted for by a corresponding increase in expressions of individualistic cultural values.

## Materials and Methods

The American English corpus and simplified Chinese corpus of the Google Books Ngram Viewer were used to track the frequency of affection words from 1960 through 2008. Given that Google Book Ngram has scanned and digitized 4% of the books published since 1800, the database is the world’s largest e-book repository (Michel et al., 2010) and is very useful for quantifying cultural change across millions of books (Greenfield, 2013). Especially from 1960 to 2000, more than 53% of books published in the United States were scanned by the Viewer each year, suggesting that the books scanned by the Ngram Viewer are fairly representative.

Our unit of analysis was the frequency of two types of affection behavior: verbal behavior (e.g., love you, like you; *ai ni*, *xihuan ni* in Chinese) and non-verbal behavior (e.g., kiss, hug; *qin wen*, *yong bao* in Chinese). We also quantified first-person singular pronouns (e.g., *I*, *me*, *my*, *mine*, *myself*) in the two languages as the measure of individualism. We then tested the changing effect of AC word use over time by examining the correlation between year and the frequency of key words. In American books, furthermore, the correlation between the use of AC words and that of individualism words was also analyzed.

Although Google has digitized 15 million books, it does not provide information on book types (e.g., fiction vs. non-fiction). Moreover, some types of books (such as novels) were often portrayed in a way that didn’t match the times depicted. We therefore took measures to rule out the influence of fiction. Specifically, we consulted the Statistical Abstract of the United States (U.S. Census, 2004) and obtained the percentage of books published each year in the United States that were fiction. These statistics were available only for 1960–2000. We used them as a control variable. For the Chinese corpus, however, we found that the Chinese Library Classification had 22 basic categories that did not include fiction as a single category; as a result, we did not obtain these data. Based on the American corpus, we found that the percentage of fictions has little impact on the overall analysis (beta weights ranged from 0.16 to 0.29).

Correlation coefficients represent the direction and magnitude of the linear relationship between the variables of interest, which in this instance were the word frequency and year. To document change from Endpoint 1 (1960) to Endpoint 2 (2008), we also included a second effect size, *d*, based on the two endpoints of the regression line divided by the standard deviation.

## Results

**FIGURE 1:**
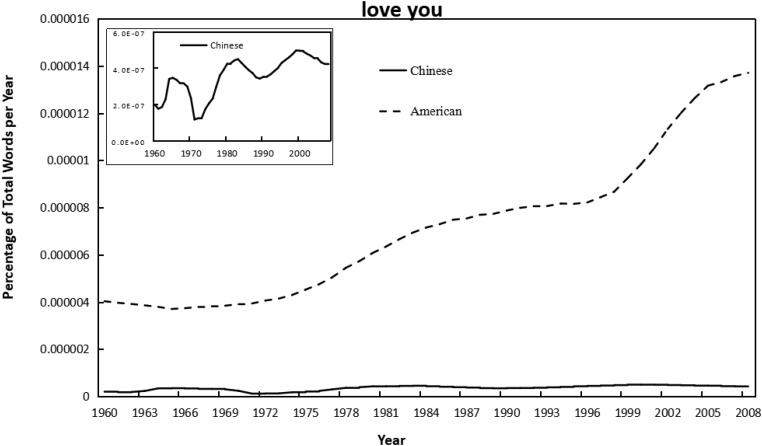
Changes in verbal phrase “love you” in Google corpus of Chinese (simplified) and American books, 1960–2008. Since there was a huge difference in the frequency magnitude of “love you” in the Chinese and in the American corpus, the smaller one (in Chinese) was presented twice, with the second shown in a little graph to reflect the trend more visible.

**FIGURE 2:**
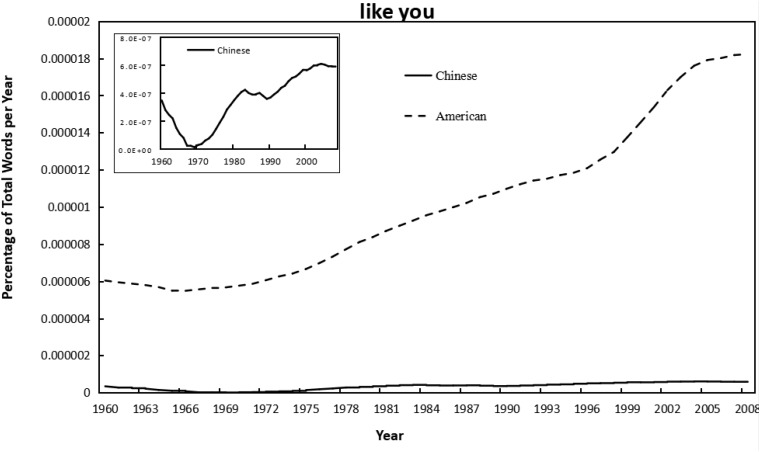
Changes in verbal phrase “like you” in Google corpus of Chinese (simplified) and American English books, 1960–2008. Since there was a huge difference in the frequency magnitude of “like you” in the Chinese and in the American corpus, the smaller one (in Chinese) was presented twice, with the second shown in a little graph to reflect the trend more visible.

**FIGURE 3:**
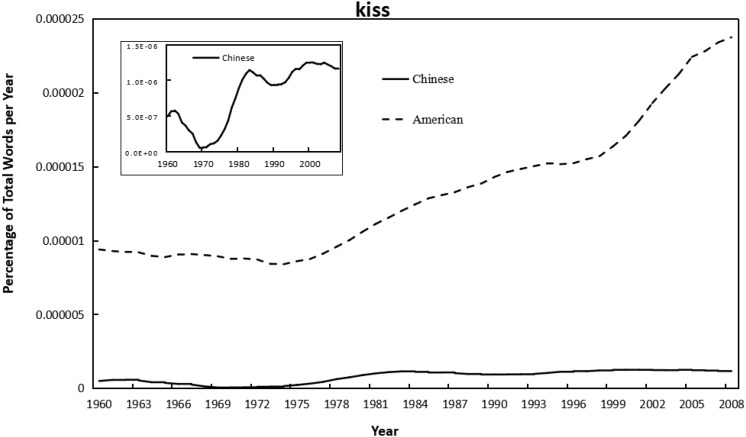
Changes in non-verbal word “kiss” in Google corpus of Chinese (simplified) and American English books, 1960–2008. Since there was a huge difference in the frequency magnitude of the word “kiss” in the Chinese and in the American corpus, the smaller one (in Chinese) was presented twice, with the second shown in a little graph to reflect the trend more visible.

**FIGURE 4:**
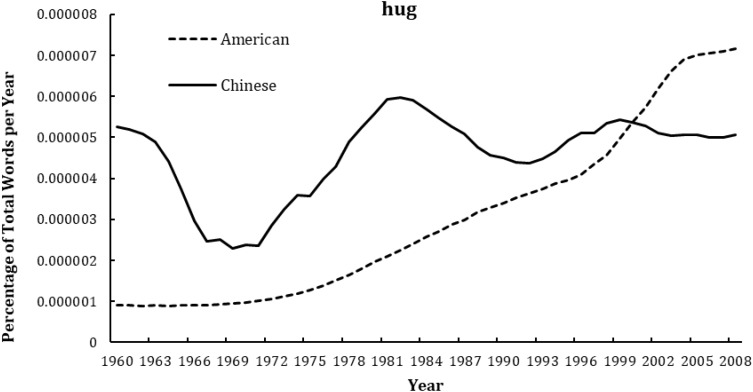
Changes in non-verbal word “hug” in Google corpus of Chinese (simplified) and American English books, 1960–2008.

As shown in Figure 1–4, the frequency of both verbal (e.g., love you, like you) and non-verbal (e.g., kiss, hug) affection words increased with year in the Chinese and American English corpus of Google books. Meanwhile, independent-sample *t*-tests showed that the frequency of using affection words in Chinese books was significantly lower than that in American books for “love you” (*t* = −15.53, *p* < 0.001, *d* = −3.14), “like you” (*t* = −16.59, *p* < 0.001, *d* = −3.35), and “kiss” (*t* = −18.91, *p* < 0.001, *d* = −3.82); but significantly higher than in American for “hug” (*t* = 4.39, *p* < 0.001, *d* = 0.89). Similarly, the frequency of using individualism-related words (first-person singular pronoun) was also lower in Chinese versus American books (*t* = −15.57, *p* < 0.001, *d* = −3.15). This suggested that, by and large, the frequency of using affection words was higher in the highly individualistic United States than in the less-individualistic China, except that the “hug” was more frequently used in Chinese. For the raw data, see Supplementary Data Sheet S1.

As shown in Tables 1, 2, correlational analyses revealed that all the affection words increased from 1960 to 2008, in Chinese books, for “love you” (*r* = 0.77, *p* < 0.001), and for “like you” (*r* = 0.71, *p* < 0.001), for “kiss” (*r* = 0.84, *p* < 0.001), for “hug” (*r* = 0.49, *p* < 0.001); and in English books, for “love you” (*r* = 0.96, *p* < 0.001), for “like you” (*r* = 0.96, *p* < 0.001), for “kiss” (*r* = 0.93, *p* < 0.001); for “hug” (*r* = 0.96, *p* < 0.001). The use of all affection words was also significantly correlated with that of first-person singular pronoun in both Chinese (*r* = 0.53∼0.89, *p* < 0.001) and American English (*r* = 0.96∼0.97, *p* < 0.001). Further, the integrated analysis of using verbal affection words, non-verbal affection words, and all affection words in both languages confirmed the linear shift over time and showed a positive relationship between affection and individualism (*r* = 0.45∼0.88, *p* < 0.001). Meanwhile, as shown in Supplementary Table S1, the linear model of affection words over year in American English books remained significant when controlling for the percentage of books that were fiction: for “love you” (β = 1.01, *p* < 0.001), for “like you” (β = 1.02, *p* < 0.001), for “kiss” (β = 1.04, *p* < 0.001), and for “hug” (β = 1.02, *p* < 0.001).

**Table 1:** Changes in the use of affection words and of individualism words in the simplified Chinese books, 1960–2008.

	*r* with year	Beta for year square	Use 1960 vs. 2008 (SD)	*d*	% change	*r* with first-person singular pronoun
Love you (*Ai Ni*)	0.77^∗∗∗^	−0.07	0.000020–0.000042% (0.000011%)	2.00	210%	0.53^∗∗∗^
Like you (*Xihuan Ni*)	0.71^∗∗∗^	0.24	0.00022–0.00027% (0.000064%)	0.78	123%	0.89^∗∗∗^
Kiss (*Qin Wen*)	0.84^∗∗∗^	−0.02	0.000049–0.00012% (0.000042%)	1.69	245%	0.73^∗∗∗^
Hug (*Yong Bao*)	0.49^∗∗∗^	−0.04	0.00053–0.00051% (0.00010%)	−0.20	96%	0.89^∗∗∗^
Verbal affection	0.74^∗∗∗^	0.20	0.00024–0.00031% (0.000073%)	0.96	129%	0.86^∗∗∗^
Non-verbal affection	0.61^∗∗∗^	−0.03	0.00057–0.00062% (0.00014%)	0.36	109%	0.87^∗∗∗^
All affection words	0.67^∗∗∗^	0.05	0.00081–0.00093% (0.00021%)	0.57	115%	0.88^∗∗∗^

*Verbal affection words include “love you” and “like you” and non-verbal affection words include “kiss” and “hug.” Usage means and d (difference in terms of standard deviations) are based on the frequency of affection words at 1960 and 2008. The Beta for year square is from a regression equation with the square of year (centered) as the dependent variable. ^∗∗∗^p < 0.001.*

**Table 2:** Changes in the use of affection words and of individualism words in the American English books, 1960–2008.

	*r* with year	Beta for year square	Use 1960 vs. 2008 (SD)	*d*	% change	*r* with first-person singular pronoun
Love you	0.96^∗∗∗^	0.23	0.00040–0.0014% (0.00030%)	3.33	350%	0.96^∗∗∗^
Like you	0.96^∗∗∗^	0.25	0.00060–0.0018% (0.00041%)	2.93	300%	0.96^∗∗∗^
Kiss	0.93^∗∗∗^	0.33^∗^	0.00094–0.0024% (0.00045%)	3.24	255%	0.97^∗∗∗^
Hug	0.96^∗∗∗^	0.27	0.000096–0.00073% (0.00021%)	3.01	760%	0.96^∗∗∗^
Verbal affection words	0.96^∗∗∗^	0.24	0.0010–0.0032% (0.00072%)	3.06	320%	0.96^∗∗∗^
Non-verbal affection	0.94^∗∗∗^	0.32^∗^	0.0010–0.0031% (0.00066%)	3.18	310%	0.97^∗∗∗^
All affection words	0.95^∗∗∗^	0.28	0.0020–0.0063% (0.0014%)	3.07	315%	0.97^∗∗∗^

*Verbal affection words include “love you” and “like you” and non-verbal affection words include “kiss” and “hug.” Usage means and d (difference in terms of standard deviations) are based on the frequency of affection words at 1960 and 2008. The Beta for year square is from a regression equation with the square of year (centered) as the dependent variable. ^∗^p < 0.05, ^∗∗∗^p < 0.001.*

In addition, in a regression equation with the square of year (centered) as the dependent variable, the quadratic model was significant only for “kiss” in American English (β = 0.33, *p* < 0.05), but not significant for all the other affection words in neither language (β = −0.07∼0.27, all *p* > 0.05). That was, the linear model was stronger than the quadratic model for all the affection words in both languages (see Tables 1, 2), suggesting a steady linear increase in using affection words in both Simplified Chinese and American English books.

## Discussion

Based on Google digitalized books, the current findings demonstrate the changing landscape of love expression in China and in the United States, in terms of both verbal and non-verbal affection words. In particular, a convergent and linearly increasing trend of AC emerged from 1960 through 2008, although the frequency of affection words was lower in Chinese than in American English books (except for the word “hug”). Further, the frequency of using affection words was positively related to that of individualism in both Simplified Chinese and American English books. These results suggest that affection exchange becomes more popular in adaptation to individualistic urban environments, both in Western and Eastern societies.

Supporting our hypotheses, with the worldwide shift from rural community/gemeinschaft to urban society/gesellschaft and from collectivism to individualism, the frequency of using affection words increased in the United States and in China. These results were consistent with previous observations, especially in China, in which the expression of love was traditionally discouraged but has become increasingly legitimized in recent decades. Although only a short period (1960–2008) was considered in this study, because of the limited coverage of the simplified Chinese (officially used since the late 1950s) and of the scanned physical books in Google Ngram (up to 2008), the increase in affection words was still significant. This period also represents the most dramatic increase of urbanization and individualization in modern times (Fukuyama, 1999).

The results suggest not only that people both in the United States and China become more open to expressing love in recent decades, but also that the increases in affectionate expression started earlier and were more pronounced in the United States than in China. This may be because increases in urbanization and modernization happened earlier in the United States than in China (Inglehart and Baker, 2000). In the United States, the acceleration of cultural change, particularly in individualism, started in the late 1960s through the 1970s when world wars ended and individualist values, such as personal rights and individual self, were emphasized (Twenge et al., 2013; Yu et al., 2015). In comparison, China has carried out a series of economic system reforms since the late 1970s, so that personal goals were ideologically emphasized and freely chosen love became an integral part of the process of marriage and family relationships (Yao, 2012), despite ongoing influences of traditional cultural values (Zhong and Cheng, 2014).

It should be noted that Google Ngram Viewer has some limitations. First of all, although Google Book Ngram scanned and digitized 4% of the books published, these books may not be randomly selected (Michel et al., 2010). Second, Google Ngram Viewer might make some mistakes in character recognition. For example, in the 18th century, early letters had a so-called “long s,” which looked similar to an “f.” Moreover, some books with an ambiguous publication year may have been incorrectly categorized with respect to their publication year, which may affect the accuracy of the results. Caution is therefore urged in the interpretation of these findings. In addition, Google Ngram Viewer may be more likely to include e-books written in English than in Chinese. Moreover, the content of the corpus is entirely derived from published books and does not include unpublished books or other forms of text, nor does it include exponentially growing networks and electronic information. In future directions, for example, cultural products such as pop songs (Dewall et al., 2011), newspapers (Nafstad et al., 2010), and social media (Wu et al., 2018) can be used to test the effect of cultural change. In addition, quantitative studies such as longitudinal or intergenerational surveys can also be helpful to verify the current findings based on qualitative analysis.

The current findings suggest several alternative interpretations. For one, the lower level of affectionate expression in rural versus urban societies does not mean that rural residents actually experienced less love than their urban counterparts. In the past, the love relationship often happened in secret, such as Zhang Sheng and Cui Yingying (characters of traditional Chinese love story: The Romance of West Chamber) (Yang, 2014), which might not be captured in published books. Men and women may like each other, but their parents may not know and may separate them. A man may love a woman but refuse to express his feelings to her, or a woman’s affection for a man may be misinterpreted (Tian, 1936). Second, it is important to acknowledge that the expression of love is not equal to the quality of love. Using individualistic words (“I” expressions) as a measure of individualism may invoke a paradox about “true” love, namely that it is the experience of “we” not “I” that looms large when one is in love. True intimacy and happiness invoke a sense of being together, whereas “I” is related to personal goals and desires (Pennebaker et al., 2003). Third, although the quadratic model for all the affection words in Chinese was not significant, the pattern of “love you” was unlike that of the other markers (see Figure 1), in which the other three patterns all decreased from 1960 to 1970, whereas “love you” increased from 1960 to 1965 and then declined. What happened in 1965 to cause this change is yet to be discovered, but the liberated Chinese people were immersed in the passion of personal romanticism (indicated by free love and women’s liberation) and revolutionary romanticism, which flourished in the 1940s and was reflected in the rise of carols and political lyrics (Shi, 2016). Therefore, the specific pattern of “love you” between 1960 and 1965 may be partially accounted for by Chinese political lyrics, in which the phrase “love you” is probably directed to the motherland or political figures rather than to one’s romantic partners. In addition, the frequency of using “hug” was the only one which was higher in Chinese than in American books. This may due to another common usage of “hug” in Chinese without any romantic sense, like “hug or embrace the future” (i.e., *“Yong Bao Wei Lai”* in Chinese) and “hug or embrace the world” (i.e., *“Yong Bao Shi Jie”* in Chinese), which was very popular in carols and political lyrics in China.

## Conclusion

In conclusion, the present research demonstrates an increasing trend of love expression in China and in the United States concurrent with a sociodemographic shift from rural community/gemeinschaft to urban society/gesellschaft. The quantitative analyses, based on Google Ngram Viewer, document the cultural change in using verbal and non-verbal affection words over past decades, both in Simplified Chinese and American English books, and this change could be explained by the increase in individualistic values. The results suggest that both Chinese and Americans are becoming increasingly open to communicating affection and love in adaptation to individualized urban societies, even though there are still considerable gaps between the East and the West.

## Author Contributions

MW designed the research. MW and CZ ran the pilot study and developed the early draft. MW, BL, and LZ wrote the manuscript. All authors participated the manuscript revisions.

## Conflict of Interest Statement

The authors declare that the research was conducted in the absence of any commercial or financial relationships that could be construed as a potential conflict of interest.
