# Supplementary material for: Culture Change and Affectionate Communication in China and the United States: Evidence From Google Digitized Books 1960–2008
Source: Front Psychol. 2019 May 22;10:1110. doi: 10.3389/fpsyg.2019.01110 (PMC6540734; doi:10.3389/fpsyg.2019.01110)
Supplement: Supplementary file 1 [file Table_1.pdf]

Table S1. *Beta* for Year when controlling for the percentage of fiction books in American Books, 1960-2008.

	Kiss	Hug	Love you	Like you	Verbal affection words	Nonverbal affection words	All affection words
Beta for Year	1. 04***	1. 02***	1. 01***	1. 02***	1. 02***	1. 04***	1. 03***

Notes. Verbal affection words include “love you” and “like you”, and nonverbal affection words include “kiss” and “hug”.
