# Supplementary material for: Culture Change and Affectionate Communication in China and the United States: Evidence From Google Digitized Books 1960–2008
Source: Front Psychol. 2019 May 22;10:1110. doi: 10.3389/fpsyg.2019.01110 (PMC6540734; doi:10.3389/fpsyg.2019.01110)
Supplement: Supplementary file 2 [file Data_Sheet_1.pdf]

***Data Sheet 1.* The frequency of using affection words and individualism words in Google digitized books 1960-2008.**

Language	*love you_frequency*	*like you_frequency*	*kiss_frequency*	*hug_frequency*	*individualism*
Simplified-	0.000000197450	0.000000343190	0.000000489931	0.000005253475	0.004070644598
Chinese	0.000000174753	0.000000274552	0.000000561732	0.000005192229	0.004032442452
	0.000000184326	0.000000240340	0.000000572031	0.000005083172	0.003919030121
	0.000000226596	0.000000214969	0.000000526165	0.000004894800	0.003478120608
	0.000000339326	0.000000147096	0.000000403993	0.000004418635	0.003010092936
	0.000000345250	0.000000102551	0.000000359449	0.000003733785	0.002407989396
	0.000000334849	0.000000075958	0.000000291067	0.000002967023	0.001535223065
	0.000000315568	0.000000018861	0.000000246205	0.000002477094	0.001084108666
	0.000000314735	0.000000018861	0.000000124928	0.000002504374	0.000549605298
	0.000000297534	0.000000008963	0.000000046959	0.000002289219	0.000369339593
	0.000000233533	0.000000024040	0.000000054377	0.000002376461	0.000527598018
	0.000000115376	0.000000031966	0.000000062303	0.000002363665	0.000604741976
	0.000000121977	0.000000056350	0.000000098879	0.000002855128	0.000584957316
	0.000000125148	0.000000071509	0.000000110249	0.000003252373	0.000709748627
	0.000000173637	0.000000098896	0.000000145105	0.000003583622	0.000677229227
	0.000000204757	0.000000140074	0.000000218637	0.000003559916	0.001081621019
	0.000000230155	0.000000184400	0.000000309542	0.000003975673	0.001262039068
	0.000000296899	0.000000226395	0.000000428818	0.000004279280	0.001568788628
	0.000000358906	0.000000278581	0.000000611298	0.000004899405	0.001988872819
	0.000000388711	0.000000311607	0.000000738871	0.000005245175	0.002450890689
	0.000000422784	0.000000345672	0.000000885550	0.000005584894	0.002860396482
	0.000000426199	0.000000376866	0.000001005785	0.000005934895	0.003290324292
	0.000000441882	0.000000404507	0.000001088405	0.000005981769	0.003303390261
	0.000000448524	0.000000419589	0.000001141266	0.000005913791	0.003253364076
	0.000000427273	0.000000396667	0.000001107054	0.000005685815	0.002955962503
	0.000000406462	0.000000385582	0.000001064042	0.000005466022	0.002834148166
	0.000000387441	0.000000388948	0.000001068147	0.000005247938	0.002635916583
	0.000000371737	0.000000397209	0.000001018701	0.000005081420	0.002475622674
	0.000000348084	0.000000375779	0.000000964013	0.000004758391	0.002265524658
	0.000000341232	0.000000354808	0.000000929737	0.000004569348	0.002112266019
	0.000000350846	0.000000364237	0.000000930162	0.000004491968	0.002054765601
	0.000000352669	0.000000386219	0.000000938556	0.000004395103	0.002130987083
	0.000000364889	0.000000405857	0.000000944562	0.000004379509	0.002216427909
	0.000000382526	0.000000434212	0.000000967586	0.000004484362	0.002388838317
	0.000000399522	0.000000448574	0.000001029661	0.000004655142	0.002558477482
	0.000000427088	0.000000481164	0.000001114951	0.000004933583	0.002810298300
	0.000000442726	0.000000504852	0.000001156897	0.000005107133	0.003003711604
	0.000000457619	0.000000514591	0.000001149449	0.000005109904	0.003067070860
	0.000000475028	0.000000535684	0.000001203905	0.000005338926	0.003186615742
	0.000000496003	0.000000562188	0.000001244501	0.000005438303	0.003270864370
	0.000000494340	0.000000558765	0.000001246851	0.000005356174	0.003213221220
	0.000000491009	0.000000572821	0.000001243890	0.000005270205	0.003189432176
	0.000000478685	0.000000594062	0.000001226162	0.000005116854	0.003127474618
	0.000000468476	0.000000598139	0.000001221346	0.000005035849	0.003044852286

	0.000000454260	0.000000605820	0.000001245320	0.000005072862	0.003033840077
	0.000000451008	0.000000599769	0.000001217447	0.000005073694	0.002977421761
	0.000000430525	0.000000588701	0.000001194026	0.000004998494	0.002917241535
	0.000000421836	0.000000586449	0.000001161753	0.000004998193	0.002926221090
	0.000000420374	0.000000582731	0.000001158689	0.000005053409	0.002915674649

Language	*love you_frequency*	*like you_frequency*	*kiss_frequency*	*hug_frequency*	*individualism*
American-English	0.000004031601	0.000006035482	0.000009388236	0.000000961852	0.005291156015
	0.000003965185	0.000005940444	0.000009281004	0.000000960427	0.005243421691
	0.000003915615	0.000005868753	0.000009224458	0.000000942905	0.005195599154
	0.000003857683	0.000005793562	0.000009196059	0.000000945931	0.005148322590
	0.000003792292	0.000005681365	0.000008955980	0.000000937698	0.005090650778
	0.000003703397	0.000005493305	0.000008872625	0.000000942984	0.005037218419
	0.000003736448	0.000005484999	0.000009050110	0.000000950735	0.005074191146
	0.000003788501	0.000005563353	0.000009078649	0.000000960903	0.005136822042
	0.000003817703	0.000005637989	0.000009011279	0.000000968190	0.005133792516
	0.000003842731	0.000005668187	0.000008927113	0.000000997725	0.005149751403
	0.000003906420	0.000005760390	0.000008759529	0.000001027686	0.005152624501
	0.000003936228	0.000005859323	0.000008777689	0.000001072920	0.005146405294
	0.000004060373	0.000006053319	0.000008708912	0.000001119021	0.005121844622
	0.000004143449	0.000006260655	0.000008412327	0.000001186366	0.005103853871
	0.000004298556	0.000006415395	0.000008390414	0.000001249141	0.005063453576
	0.000004523710	0.000006658907	0.000008592976	0.000001353044	0.005101043410
	0.000004751153	0.000006975973	0.000008740024	0.000001457706	0.005132987938
	0.000005052106	0.000007327545	0.000009107827	0.000001592314	0.005217466496
	0.000005448570	0.000007726432	0.000009563074	0.000001739749	0.005303373998
	0.000005728212	0.000008110907	0.000010021277	0.000001885638	0.005401769171
	0.000006077443	0.000008380943	0.000010588489	0.000002045662	0.005452948127
	0.000006350049	0.000008712762	0.000011121318	0.000002180144	0.005487398266
	0.000006665029	0.000008989548	0.000011554223	0.000002331986	0.005538623867
	0.000006940190	0.000009265672	0.000012018680	0.000002497114	0.005596147232
	0.000007157192	0.000009553308	0.000012445423	0.000002663377	0.005662263051
	0.000007297973	0.000009762762	0.000012849353	0.000002801514	0.005730777061
	0.000007486150	0.000009985250	0.000013064749	0.000002961700	0.005771660057
	0.000007539825	0.000010216567	0.000013264295	0.000003082146	0.005821621760
	0.000007693745	0.000010526928	0.000013605040	0.000003273943	0.005930398469
	0.000007733789	0.000010708960	0.000013843476	0.000003390251	0.005986986140
	0.000007877380	0.000010980308	0.000014291080	0.000003502846	0.006049228057
	0.000007989662	0.000011222668	0.000014635991	0.000003624565	0.006086444551
	0.000008059034	0.000011420545	0.000014836822	0.000003741150	0.006113508361
	0.000008064357	0.000011517396	0.000015022454	0.000003836064	0.006159177832
	0.000008168233	0.000011713314	0.000015211174	0.000003972317	0.006230281707
	0.000008158815	0.000011840849	0.000015159687	0.000004076080	0.006268362414
	0.000008217127	0.000012083614	0.000015222675	0.000004206722	0.006359938017
	0.000008442713	0.000012554073	0.000015492760	0.000004458365	0.006536113387
	0.000008671935	0.000012959147	0.000015712092	0.000004673532	0.006716398747
	0.000009243552	0.000013748096	0.000016362968	0.000005083783	0.007010994158

0.000009833711	0.000014555041	0.000017091234	0.000005487144	0.007300945810
0.000010520419	0.000015377471	0.000018114194	0.000005853919	0.007676908472
0.000011334383	0.000016286507	0.000019298269	0.000006315981	0.008057837670
0.000012019486	0.000016997096	0.000020306905	0.000006745291	0.008380534322
0.000012633181	0.000017601395	0.000021251610	0.000007045335	0.008776965670
0.000013166687	0.000017911411	0.000022420895	0.000007140890	0.009267272847
0.000013322460	0.000018011750	0.000022810725	0.000007178790	0.009428388566
0.000013576301	0.000018174198	0.000023400939	0.000007222970	0.009650539395
0.000013711496	0.000018228061	0.000023748200	0.000007290071	0.009784005443
